# From a Technology That Replaces Human Perception–Action to One That Expands It: Some Critiques of Current Technology *Use* in Sport

**DOI:** 10.1186/s40798-021-00366-y

**Published:** 2021-10-23

**Authors:** Carl T. Woods, Duarte Araújo, Keith Davids, James Rudd

**Affiliations:** 1 Institute for Health and Sport, Victoria University, Melbourne, Australia; 2 CIPER, Faculdade de Motricidade Humana, Universidade de Lisboa, Lisbon, Portugal; 3 Sport and Human Performance Research Group, Sheffield Hallam University, Sheffield, UK; 4 Norwegian School of Sport Sciences, Oslo, Norway

**Keywords:** Sports technology, Ecological dynamics, Device paradigm, Knowledge of/about, Experiential knowledge, Direct perception

## Abstract

Information technology has been integrated into most areas of sport, providing new insights, improving the efficiency of operational processes, and offering unique opportunities for exploration and inquiry. While acknowledging this positive impact, this paper explores whether sufficient consideration has been directed towards what technology risks detracting from the learning and developmental experiences of its users. Specifically, viewed through the philosophical lens of the device paradigm, and considering a more ecological account of technological implementation, we discuss how technology *use* in sport could subtly disengage educators and applied sports scientists from performance environments. Insights gleaned from such an ecological account of technology implementation could lead sports science and educational teams to ask and reflect on tough questions of current practice: i.e. *has too much control been given to technological devices to ‘solve’ problems and communicate knowledge (about) in sport? Has technology improved the skills of players and performance staff? Or are performance staff at risk of becoming over-reliant on technology, and as a result, reducing the value of experiential knowledge (of) and intuition?* Questions like these should be asked if technological devices, purported to support aspects of practice, are continually integrated into the sporting landscape.

## Key Points

- This position explores what technology use and implementation in sport risks detracting from the learning and developmental experiences of its users.
- It views technology use through a philosophical lens – proposing that without careful consideration, technology use in sport could risk subtly disengaging educators and applied sport scientists from their performance environments.
- We propose a more focal appreciation of technology through the framework of ecological dynamics – offering examples from high-performance sport and physical education to show what such a focality could look like, compared with current modes of indirect, data-driven compliance.

## Introduction

Our future is a race between the growing power of our technology and the wisdom with which we use it. Let’s make sure that wisdom wins – Stephen Hawking

Technology has been embedded into most regions of the performance landscape characterising sport and physical education [1]. For the most part, this integration throughout history has led to some considerable breakthroughs, many of which—like the use of carbon fibre in the design of road bikes, polyurethane in swim suits or automated motion tracking sensors—have advanced athletic performance and preparation [2]. Further, information technology (which is the concern of our article) has enhanced interventions intended to support physical activity and inclusion at all levels of sports participation, thereby leading to positive behavioural changes [3]. More specifically, in high-performance contexts, technology use can purportedly help practitioners monitor and track training interventions, assist with decision making around the selection and development of athletes, and analyse team or individual player behaviours in minute detail to help devise strategies intended to win competitions and championships [4–7]. At the other end of the performance spectrum, in physical education, technology can be used to enhance engagement, motivation, quality, and inclusion, assist educators with pupil assessment, support activity, and track core competencies of development (physical, affective, social and knowledge, and understanding) [3, 8–10].

While acknowledging its positive impacts, this paper questions whether sufficient consideration has been directed towards what information technology implementation might detract from the learning and developmental experiences of its users. More specifically, we explore the subtle ways technology risks disengaging its users from their performance environments, leading to a progressive over-reliance on a device to continually inform their (indirect/mediated) perceptions *about* things—deciding *for* them on what to do. This paper is intended to encourage sports practitioners to reflect upon how they *use* technology, drawing upon various philosophical critiques of technologies in Western society to help guide this narrative. Echoing the sentiments of Norman [11], this work should not be read as being ‘anti-technology’, but rather ‘pro-human’. It should be seen to be raising awareness of how technology implementation and use across the sporting landscape risks altering a practitioner’s agency, overemphasising compliance, conformity, abstraction, and cause and effect practices that may distance them from a performance environment, and at worst, remove them all together.

## A (Brief) Philosophical Excursion

### (Dis)engagement, (Over)reliance, Compliance and Conformity, and a Fear of Uncertainty

In contemporary Western society, the technological evolution is typically seen as the hallmark of intelligence and complexification, a type of triumph that ontologically sees society attempt to exert ever-increasing control over the constraints of the environment [12]. This control over context, as highlighted by Reed [13], can be traced to a deeply rooted societal fear of uncertainty, which can be attributed to the dualistic philosophy of Descartes. Progressively, this control over context has expanded to a control over people. Manifest in contemporary society, for example, the fear of uncertainty has led to a rise of managerialism in the marketplace, in which technology and automatisation are used to replace ‘fallible’ human performance and judgement with repeatable ‘if–then propositions’ and rapid decisions made by devices and instruments [13]. Resultantly, people have grown to degrade first-hand, direct experiences [13], in favour of a strict compliance with ever-increasing specification, rules, regulations, and conventions established to commodify prescribed experiences and products, centralising market certainty, deskilling people in the process (note, this can be detected in phrases, like ‘the computer won’t let me do that’ or ‘the system is down’). Thus, while current technological evolution has improved many aspects of our lives, it is an evolutionary work in progress, rooted in a mechanistic, Cartesian worldview, founded on a fear of uncertainty [13, 14].

While valuable in places, such a worldview continues to drive a subtle divide between person and place, mediating direct interactions of an individual with an environment and replacing first-hand, experiential knowledge and expertise in favour of directions, conformity, abstraction, and a (false) sense of certainty [12]. The rise of the continued quest for managerial certainty in the workplace is what Reed [13] refers to as the *machining of the mind*:

[A]s information technology is integrated into workplaces, the information available to workers, and the actions they are *allowed* to take on the basis of that information, comes increasingly *from* computers and programs, not through the workers’ own understanding of situations. (p. 65, our emphasis)

Concerningly, this mediated interaction can be observed everywhere in contemporary Western society. From noting the number of people with their heads down looking at their ‘smart’ phones while waiting for a takeaway coffee ordered from the local café (perhaps even being ordered ‘online’ beforehand, thereby mitigating the need for a direct encounter with the barista), to the weekend hiker who passively follows the ‘optimal’ route that has been ‘mapped out’ in advance *for* them by a global positioning satellite (GPS) sending coordinates to a ‘smart’ watch on their wrist. What these, and many other everyday examples demonstrate is that we are fast becoming a society that is ‘allergic’ to emergent unpredictability, uncertainty, and variation in the environment, perhaps too willingly giving control to technological devices and programmes to engage and correspond with our surrounds indirectly *for* us [13]. That is, we are more inclined to attend to the digitised coordinates on a ‘smart’ watch, rather than to the rhythms of the hiking trail!

Growing social fears of uncertainty are in stark contrast to what the eminent philosopher John Dewey called for over 100 years ago when arguing for primary, first-hand experience. For Dewey, experiencing the world was not about mechanically or reflexively following a script laid out for us in advance, but rather about directly perceiving and discovering what the world actually means for us by undertaking practical, everyday tasks. Indeed, such practical, first-hand experience can be risky and even lead to performance ‘failures’, but it is precisely through this experience that knowledge grows, discoveries are made, and learning emerges [13]. The counteraction to fearing uncertainty was captured by Samuel Beckett, the Irish writer, in the famous paragraph 6 of his 1983 story *Worstward Ho*, with the much-quoted lines: ‘Ever tried. Ever failed. No matter. Try again. Fail again. Fail better’.

This alternative view of uncertainty and failure in contemporary society highlights the value of direct experiences with performance environments in life, from which information technological implementation may be shielding humans. Ingold [12] argues, for example, that rather than being a process of complexification and intelligence,^1^ the modern technological evolution is akin to an externalisation or objectification, replacing the etymological connotation of *tekhnê*—as one of skilled craftspersonship centred around environmental engagement—and *mêkhanê*—as the dextrous movements of a skilled craftsperson—with one based around de-humanised, mechanised, and executable processes, analogous to those seen in factory production lines. Simply, Ingold’s [12] arguments are placed in a worldview that sees contemporary technology as something that detaches person from place; a sentiment captured by phenomenologist, Malpas [15]: ‘under the reign of technological modernity, our relatedness to place is not obliterated, but is rather covered over, ignored, made invisible’ (p. 63). Indeed, despite these somewhat pessimistic views of technology by both Ingold [12] and Malpas [15], it is important to note that it can support and actually promote positive behavioural change in certain instances [e.g. 3, 16]. Our point, however, is that there are many unquestioned assumptions and consequences of the technological trend to replace and intervene in direct human perception–action interactions with the environment—some of which we discuss here. For example, a particular emphasis on artificial intelligence in sport (for detailed critique, see Araújo et al. [17]) is already proclaimed as the key contributor to the fourth industrial revolution [18].

This progressive objectification is captured in Borgmann’s [19] philosophical critiques of contemporary technological devices. Interestingly, despite the rate at which technology in sport has proliferated in recent years [20], such philosophical critiques of technological devices are yet to be considered in detail by applied sports scientists and physical educationalists. This is a concern, as at the heart of Borgmann’s [19] contention is the proposition that technology subtly alters the ways in which humans engage with each other and the environment. Specifically, captured within the device paradigm, Borgmann [19] describes the patterns that emerge from societal interactions with contemporary technologies, proposing that such devices have profound (yet subtle) influences on the way (Western) societies, communities and groups take up with the world. More directly, he argues that contemporary technological devices separate users from the environment through their consuming undertones and abstracted designs.

While acknowledging the benefits of technologies, Norman [11] raises comparable concerns. He argues that technology risks entrapping individuals through their overly mechanistic and de-humanised designs. This entrapment can be attributed to ‘design error’, suggesting that most technologies are developed without careful consideration of its users’ capabilities (or limitations). More directly, the design of technology is often done in such a way that promotes compliance and conformity, a blanketing approach that likely underestimates the experiential knowledge of the individual using it. To remedy this weakness, Norman [11] calls for a more humanised view of technology, one which would maintain the fundamental benefits of technologies, like freeing up time for more direct involvement in interpretation, synthesis, and exploration, but not lure them into simply conforming to the information they prescribe. Accordingly, more functional technology would have specific and identifiable applications, but concurrently open new lines of inquiry beyond the boundaries of the information they provide [11, 12]. This more humanised view of technology challenges its subtle entrapment by encouraging users to actively reflect upon the information provided—supporting, extending, or developing experiential knowledge. Simply, technological devices viewed through a more experientially supportive lens would become ‘instruments of revelation’ (i.e. opening new lines of inquiry to follow), as opposed to ‘instruments of control’ (i.e. driving rote repetition and conformity) [12, p. 320]. Why this is important, is that with the illusion of free time, technology is developing in a way that empties humans from their active, direct, first-hand experiences of the world, leading them to become passive, second-hand, technologically mediated *experiencers* of it [21]. This sentiment is captured eloquently by Rebecca Solnit in her exceptional book, *Wanderlust*, where she explores a history of walking:

The multiplication of technologies in the name of efficiency is actually eradicating free time by making it possible to maximise the time and place for production and minimize the unstructured travel time in between. New timesaving technologies make most workers more productive, not more free, in a world that seems to be accelerating around them. Too, the rhetoric of efficiency around these technologies suggests that what cannot be quantified cannot be valued [22, p. 10]

Here, we extend these philosophical critiques, viewing them through a Gibsonian inspired differentiation of knowledge—*of* and *about* the world—in ecological psychology [23, 24]. According to Gibson [22, 23] (and later applied to the sporting landscape by Araújo et al. [25] and Woods and Davids [26]), to know ‘about’ the environment is to indirectly perceive it, gaining a type of abstract knowledge that is shared through a variety of different represented formats (i.e. in pictures, symbols, or more aptly given our paper, in *informatics* that display how far or fast an athlete has run). Reed [21] proposed that the role of this type of knowledge is to make others aware ‘about’ things, but importantly, are not the things themselves [12]. Comparatively, knowledge *of* the environment is exemplified by directly perceiving opportunities for action that are present in it [23, 24]. It is knowledge that captures an interactive entanglement between an individual and their environment. This type of knowledge is not represented in pictures, symbols or numbers, as it is the type of knowledge that makes such things possible [21]—that is, ‘one has to have experiences *before* they can be shared’ [13, p. 2, our emphasis]. Simply, it requires an individual to directly engage with and understand a performance environment, i.e. to functionally be defined by such an environment in terms of perception–action, in an encounter that is developed by actively exploring and interacting within one’s niche. Next, we show how these Gibsonian ideas of knowledge align with those of commodities and focal things elaborated on by Borgmann [19] within the device paradigm.

### Commodities and Focal Things

Commodities can be understood as ‘highly reduced entities’ that only offer abstractions about the environment given they are ‘free of local and historical ties’ [19, p. 81]. In this way, Borgmann [19] views most contemporary technological devices as commodities in that they enable an on-demand consumption, where such devices risk separating their consumers from the physical environment is through their concealment of the mechanisms that enable the production of a commodity. The example Borgmann [19] draws upon is in the comparison of central heating and wood fires. While central heating enables relatively instantaneous warmth at the push of a button, its heating properties are commodified through the concealment of how it actually ‘works’. Stated differently, it offers a ubiquitous platform by which its users do not need to understand how it operates and/or derives heat. This view echoes Norman’s [19] sentiments about internally represented technology, which consists of devices designed in such a way they do not enable users the capability to discern how they function, leading to abstracted and superficial knowledge that promotes conformity. Here, we argue that technological devices used in commodifying ways could thus promote the development of what Gibson [23, 24] referred to as knowledge ‘about’ the environment.

In contrast, a wood fire requires an active agent to interact with an environment for it to function (i.e. through chopping wood, sparking it, and sustaining its heat). Borgmann [19] argues that because of this interaction, ‘things’ such as wood fires, engage users, both environmentally and societally, in more focal ways by directing their attention towards how and why heat (in this example) is derived. This is similar to what Norman [19] refers to as surface represented technology, which consists of devices that enable users to identify how they work by observing their physical properties, thereby directing the focus on the engagement between the ‘thing’, its function, and the environment. It is why Borgmann [19, p. 81] proposed that focal things ‘engage us in so many subtle ways that no quantification can capture them’. Accordingly, viewing technological devices in such a humanised and focal way leads to an engagement with, and connection to, more than just the information (i.e. heat) they offer. For this reason, we argue that technological devices used in such a way could support the development of their user’s experiential knowledge ‘of’ the environment—that is, their opportunities for (inter)action [23, 24].

## Implications for Technological Devices in Sport

We now explore what these philosophical critiques mean for the use of technology in sport, focusing on the subtle ways it risks disengaging its users from their performance environments through their commodifying and abstracted undertones. To support this exploration, we encourage readers to reflect upon how they currently use technology, asking themselves questions, like: *Have we unwittingly given too much control to technological devices to ‘solve’ problems and communicate knowledge? Has the integration of technological devices made us ‘more skilled’ practitioners and researchers? Or, has it led us to abandon first-hand experiential knowledge and ‘feel’? That is, have we become over-reliant on a device to tell us ‘about’ where we are going and how to find our way?* While we do not intend to explicitly answer these questions here, we do argue that simply pondering them could prove to be enriching for sports practitioners when using technological devices in their contexts.

An important point to highlight here is that while our arguments are inspired by Borgmann’s [19] propositions, we do offer a slightly different perspective when applied to sport. While Borgmann’s emphasis was on the *design* features of technological devices that risk separating or disconnecting individuals from their environments, our ideas are aligned with those of Reed [13] who contended that it is their societal *use* that risks disengagement, conformity and over-reliance, if not appropriately considered:

The problem is not with information technology, which has many wonderful uses. The problem is that our culture has succumbed to a narrow managerial perspective concerning those uses. […] Instead of using our information technology to create work-places within which human experience can grow and thrive, we are using this technology to manufacture jobs that are often little more than glorified pigeonholes. (p. 64)

It is our contention that the implementation and use of technology in sport risk the same conformity and mediation as it does in other parts of Western society. One, for example, does not have to look far to encounter this, detected in high-performance organisations that offer internships or even graduate jobs that focus (or ‘pigeon hole’) on analysing and processing copious volumes of data extracted from technological devices—of which the candidate rarely (if ever) steps directly onto the performance arena! Indeed, the processing of such data often extracted from motion tracking devices can lead to unique insights into team and individual performances, as noted by the rise of *the science of moving dots* seen in sporting competitions like the National Basketball Association [27]. We wonder, though, how many of these positions actually encourage the candidate to share their experiential perspectives on a phenomenon or topic, or whether they are simply prompted to engage through ‘a looking glass’ with questions like ‘what does the data *tell* you’? Accordingly, like Norman [11], we call for a more humanised view of technology use in sport, complementing, not replacing, a practitioner’s experiential knowledge. In this way, we are not suggesting that practitioners (i.e. coaches, applied sport scientists or educationalists) need to focus on the nuances of the micro-engineering in a device’s design. Rather, we argue that they should appreciate how its *use* could lead to over-reliant, managerial, conformist, disengaged, and inadvertently, mechanistic outlooks.

To exemplify, the use of integrated technology in sport (i.e. heart rate monitors, and motion tracking devices) has assisted with the quantification and validation of various team and individual movement patterns, the assessment of differences between training and competition demands, and the measurement of physiological and metabolic responses to various training interventions [28, 29]. Moreover, motion tracking technology, like GPS, has revolutionised sports performance analysis [30], automating a process that would otherwise be incredibly laborious, requiring hours of manual notation. However, because of this development, its use can risk being situated in a de-contextualised way through the provision of on-demand and abstracted information *about* things like an athlete’s location, distance run, and velocity reached. Indeed, while this information is of considerable use for practitioners, it is important to consider whether such devices have altered direct observations and interactions with the environment in sport—perhaps exemplified in the common, anecdotal mutterings of: ‘they aren’t working hard enough’, from coaches viewing only the numerical outputs of an athlete’s GPS report. Comments such as these, we contend, are typically made after only looking at indirect indices in the shape of performance statistics, in the absence of context—which is everything from an ecological perspective [31]. This approach may indicate an over-reliance on the device to inform a coach’s perceptions with knowledge *about* an athlete’s involvement during game play. Moreover, it is this (over)reliance that risks separating the coach from context, dampening their experiential knowledge or ‘feel’, choosing to (wittingly or unwittingly) give control to the device to shape their (indirect) perceptions about some putative state of the performer (for an example of this outside of sport, see [32]). A more extreme position happens when, flooded by data from such devices, practitioners claim for decisions about what to do, a pressure that could lead them to give up from their agency and simply conform.

Comparatively, the use of a technological device in a more focal way serves to better unlock experiential knowledge, taking its user beyond simple abstractions. Linking this back to our prior example, a GPS device used more as a focal thing that functions concurrently to a practitioner’s experiential knowledge, would not just commodify distances or locations, but would foster a platform by which its users could directly engage in an active exploration to understand why and how such information may have emerged. With respect to sport performance, the *means* (i.e. why and how) would be the focus of the device, not just its *ends* (i.e. distances, locations, heart rates, or velocity thresholds). Indeed, we are not arguing that the *ends* are any less important for sports practitioners, as they enable insight into phenomena not easily detected through direct perception (e.g. heart rate or spatial positioning of an athlete). But shifting focus towards the *means* encourages the sports practitioner to constantly question the second-hand information commodified by the device, pushing back on conformity, actively reflecting upon what the information is *showing* (note, not *telling*) them. In doing so, practitioners would be drawn into engaging with the environment (inclusive of the athlete) to better understand the means *and* ends. This view would support, extend and/or challenge the practitioner’s knowledge *of* the environment, encouraging them to be attentive to important features of the environment in situ, thereby progressing away from an over-reliance on the device to inform just their knowledge *about* the environment, documented ex situ. We elaborate on these ideas in the next section, situating technological use within a theoretical framework that views behaviour at this performer-environment scale of inquiry.

## Technology Used as a Focal Thing in Sport

Ecological dynamics offers the sporting landscape a transdisciplinary framework for understanding skill, performance, and development [33–37]. Learning, in this framework, is understood as a progressively attentive^2^ process, where perception, knowledge, and skill are not separated from context, experience, or culture [37]. Learning to become skilled, then, is a task-oriented process, requiring an embodied-*embedded* systems perspective [37]. This approach is based on continuous engagement and an active involvement with the environment, in which an individual ‘watches, listens and feels as they work’ [38, p. 179]. This component is what we contend could help dissolve disengaged and over-reliant behaviours (if noted) in technology use in sport, enabling it to be situated in a more focal way, supporting the knowledge *of* its user. So, what would this actually look like in practice?

Before answering this question, we should appreciate that to ‘educate’ within an ecological dynamics framework is not to simply *tell* someone ‘about’ something (using declaratively explicit sources of knowledge), but is to lead someone out into the world, co-designing opportunities that support emotional, embodied engagement, and cognitive, perceptual and physical interactions embedded into context (for a detailed description of education within an ecological dynamics framework, see [39]). This is an important appreciation for the intentions of this paper, as it directly implicates how practitioners would implement and use technology in a more focal way, irrespective of whether they inhabit the high-performance or physical education ends of the sporting landscape. More directly, technology usage within this framework would enable focality by guiding the attention of practitioners, athletes, and students towards the perception of the most task-relevant affordances (i.e. opportunities for action [24]) available in the environment.

Accordingly, in the following sections, we present two examples from various ends of the sporting landscape, showing how technology used in a more focal and humanised way can support the development of both practitioner and performer experiential knowledge in high-performance and physical education settings. In the first example, we show how technology use can open lines of inquiry between a coach and athlete with regard to the direct perception of task-relevant affordances. This focality, we propose, can support the co-design of enriched practice tasks, thereby supporting player performance. In the second example, we demonstrate how the innovative use of movement sonification can support the enriched movement exploration and creativity of children within a physical education setting. The focality of such technological use, we propose, can support the development of a child’s information-movement coupling, thereby enriching motor learning and development. Despite being at opposite ends of the sporting continuum, the thread binding these examples is that technology use supports direct engagement and interaction between agents and their environments—promoting the development of experiential knowledge.

### Sporting Example 1: Shifting the Focus of Technology use in High-Performance Sport to Support Practice Task Design

In this example, an experienced football coach has designed a training activity with the intention of strategically challenging a team’s offense. Constraints relating to time and athlete overloads have been used to challenge an attacking team, tasked with moving the ball down the field in an attempt to score a goal. Given this activity is played on a full-sized field with fewer players than in a competitive match, the coach anticipates that—among other things—players on the offensive team will engage in varied running efforts in an attempt to probe and exploit the constraints manipulated towards the achievement of the task goal. That is, given more space has been afforded to them relative to what is experienced within competition, the coach anticipates that players should engage in more exploratory activity when moving the ball down field—activity likely captured by data from a GPS device.

During the practice task, however, the coach has noted from the live outputs of a GPS device, coupled with their attentive observations of the practice task, that an athlete is covering considerably less distance than expected, observing that they are primarily remaining in one field location, thereby not actively and strategically exploring their surrounds. Instead of conforming to this information in a more traditional, commodifying and deductive way by perhaps just interpreting the data as evidence that the player is *‘simply not working hard enough’*, the coach follows the inquiry opened up by the data and their observations, exploring why this athlete behaviour has emerged. Such focality would likely prompt them to engage with the environment by asking the athlete questions, such as:

- Where are you positioning yourself during the activity?
- Why are you positioning yourself ‘there’?
- Are there other locations of the playing area that you might explore?
- What features of the activity are shaping this for you?
- What features could we add into the activity to encourage you to explore your surrounds and challenge your behaviours?

The point of asking such questions is to better understand the athlete’s decision making through perceptual attunement to the affordances that are (not) perceived and realised during the task. By then educating their attention towards such things in situ, the coach deepens their knowledge *of* the performance environment and how the athlete interacts with its emergent opportunities. In this sense, the unexpected movement profile of the athlete, as identified by the GPS device and supported by the coach’s attentive observations, would invite further engagement and inquiry through questions like those described above, not an ‘ends’ that closes it off in a conforming, compliant, abstracted, and objectified way. Elsewhere, we have proposed this to be a type of *abductive*, as opposed to deductive, reasoning to inquiry, encouraging sports practitioners to follow the lines of inquiry where they take them, as they emerge, when seeking to better understand a phenomenon [40]. Such abduction from the coach may even reveal that the player discovered effective ways of performing the task, and not that they were ‘simply not working hard enough’. Moreover, by viewing the information offered by the GPS device through such a focal lens, situated in a theoretical framework that promotes variability of individual-environment interactions, the coach (and athlete) would likely be presented with richer opportunities to continually (re)design the activity—designing in critical constraints that channel or challenge the athlete’s attention and problem-solving, thereby encouraging skill adaptation in future versions of the task [41].

### Sporting Example 2: Shifting the Focus of Technology use in Physical Education

Children in countries all over the world are not considered to be sufficiently active [42–44]. It is, therefore, unsurprising for policy makers to advocate the necessity of physical education in schools to tackle childhood obesity and declining levels of physical activity [44]. In collating this knowledge, technological devices, such as pedometers and accelerometers, have been proposed as the key for informing teachers and researchers *about* activity data, such as the total number of steps achieved or the time spent in target intensity thresholds. Unfortunately, however, the generation of target-driven frequency and duration information about a child’s physical activity has, for many, been counterproductive [45, 46]. Specifically, rather than this information being embedded into context to inform or tweak rich, movement-based education curricula (thereby demonstrating focality), it has led to a rise in physical education syllabi that mechanistically drive participants to reach pre-determined step counts, temporal duration thresholds or arbitrary running distances (thereby demonstrating commodification and conformity) [46]. While such programs may fulfil government targets that have been informed by data commodified by a device, they are unlikely to transform a child’s movement experience given their abstracted and deterministic undertones [45]. So, what other technological options could practitioners use, if eager to move beyond commodification of physical activity and actively support a child’s physical *education*?

Movement sonification could offer such an innovative and cost-effective solution. To facilitate this, accelerometers could be placed on pupils’ joints (e.g. wrists and ankles) to sonify movement acceleration, difference in acceleration between body parts, or parts of the body involved in the movement (for detailed explanation see [47, 48]). Accelerometers, then, (perhaps already used to commodify physical activity) would be repurposed such that schools would face no extra financial cost. In brief, sonification via accelerometry involves the calibration of a movement parameter to sound and depending on how the specified movement parameter changes, the sound changes its characteristics [49]. For example, a sound tone is triggered when a joint angle exceeds a certain velocity threshold, or a music melody is progressively distorted in reference to the amplitude of a joint angle increase [50]. Given the nature of perception and action that considers our sensory system as a whole [36, 37], there is an inherent coupling between movement and sound [50, 51]. Importantly, movement sonification does not necessarily shape novel affordances, but invites performers to explore undiscovered areas of the movement landscape, encouraging a broad range of new movements that grow their experiential knowledge, which in turn, creates new information for them to pick up.

To integrate into a physical education syllabus, a teacher would first need to calibrate the sonification equipment (movement sensors/accelerometers) to a child’s typical dance movement parameters, providing a baseline but unique movement signature. They would then manipulate movement parameters (i.e. frequency and amplitude) to certain thresholds that might be at the edges of the child’s current performance capabilities, helping them to perceive currently available affordances. An example of this would be a teacher observing a child’s irregular music-movement tempos, encouraging them to explore slower paced tempos as they dance. The teacher would calibrate the sonification system to distort or change the music when a child begins to explore such tempo. From the child's perspective, this would afford control of their learning, educating their attention through an exploration of the movement landscape that unfolds as they move. Thus, this brief example shows how innovative technology use could transform the movement experiences of children in physical education when situated in a more focal and humanised way, supporting the experiential knowledge of both the child and educator. Simply, it captures the essence of what Masschelein and Simons [52] emphasised regarding the use of technology in the school—noted within their book, *In defence of the school*:

Scholastic technologies […] are by no means tools that, when used correctly, produce well-formed young people, like finished products off an assembly line. […] Scholastic technologies are techniques that engage young people on the one hand and present the world on the other; that is, they *focus attention* on something. (p. 57–58, our emphasis)

## Conclusion

Indeed, there is much to admire in the designs and contrivances of information technologies used in sport and current opinions as adopted in our position here should not be construed to lessen them. Rather, they should highlight the subtle ways in which technology risks de-contextualising coaching and athlete-support behaviours, separating practitioners from the environment through their commodifying, managerial, conforming, and consuming undertones. Confronting these challenges through reflective questions, like those posed earlier, may permit a better understanding of how technologies evolve and how their slight realignment in use through a more focal lens could better support and engage practitioners, athletes, and students in sport. Further, viewing technology use in sport through a more relational lens may open the door to the design and integration of new technologies that harness the experiential knowledge of practitioners, athletes, and students through the information they offer—expanding their perception–action coupling by minimising any individual-environment separation technology use risks creating. Accordingly, a more focal appreciation of technology may not only enrich the use of current devices in sport, but may lead to the development of yet to be conceived devices; a prospect which we feel is particularly exciting for the future of sports technologies.

## Data Availability

Not applicable.

## Footnotes

1 Given the rise of information technology, where ‘certainty’ can be transmitted instantly into (mechanised) minds from ‘smart’ devices, Western society typically associates intelligence with adjectives like *rapid*, *reliable* and *repeatable*. This, clearly, implicates how we educate in the West. Interestingly, though, as Reed [13] points out, in some non-Western societies, ‘intelligence’ is associated with adjectives like *slow*, *careful*, and *active* – things which bode to first-hand experience and direct exposure.

2 Following Gibsonian ideas, attentive means ‘picking up’ information in the environment of use to directly regulate action.
